# An Aggressive Liposarcoma Presenting as a Perforated Colon Mass

**DOI:** 10.7759/cureus.13808

**Published:** 2021-03-10

**Authors:** Rebecca Innes, Sylvester Paulasir, Lesley Combs

**Affiliations:** 1 Surgery, Henry Ford Health System, Jackson, USA; 2 Acute Care Surgery, Henry Ford Health System, Jackson, USA

**Keywords:** liposarcoma, colon perforation, colon cancer

## Abstract

Dedifferentiated liposarcoma (DDLS) is a high-grade sarcoma that usually arises from a well-differentiated liposarcoma, which most commonly presents as a retroperitoneal mass. DDLS involving the colon is extremely rare, and only a few cases have been reported. We present a case of a DDLS that was found in the cecum and adjacent mesentery. This aggressive sarcoma developed within six months based on computed tomography (CT) findings and initially presented as a perforated colon mass. The patient was taken for emergent exploratory laparotomy including right hemicolectomy with en bloc resection. There was no metastatic disease at time of presentation, but at three-month follow-up, CT scans demonstrated metastatic disease to the liver, lungs, and multiple peritoneal implants. This case highlights a rare form of colon cancer and its aggressive nature of progression.

## Introduction

Sarcomas are a rare group of malignant tumors of mesenchymal origin that comprise less than 1% of all adult malignancies. There are many different histological subtypes based on the origin of cell type including liposarcoma, synovial sarcoma, leiomyosarcoma, rhabdomyosarcoma, fibrosarcoma, and angiosarcoma. Liposarcomas appear to arise from adipocytes. These are most commonly found in extremities and retroperitoneum [1]. Aggressiveness and metastatic potential are based on the differentiation of the tumor. There are three main morphologic subgroups: well-differentiated/dedifferentiated, myxoid/round cell, and pleomorphic liposarcoma [2]. Dedifferentiated liposarcoma (DDLS) is typically a high-grade sarcoma that arises from well-differentiated liposarcoma and usually presents as a large mass in the retroperitoneum [1].

## Case presentation

A 65-year-old Caucasian male with past medical history of alcoholism, kidney stones, and bleeding gastric ulcer presented to the emergency department of a large community hospital with right lower quadrant abdominal pain that had acutely worsened. He had low-grade pain for two weeks and associated nausea. The patient had noted progressive fatigue but denied abdominal fullness, blood in his stool, dark tarry stools, and early satiety. He did note 15-20 pound weight loss in the past year. His past surgical history included an appendectomy, cholecystectomy, and esophagogastroduodenoscopy (EGD) with intervention for bleeding gastric ulcer. The patient reported he had a colonoscopy in the past but this was unable to be confirmed and no records were found in our electronic medical record of this procedure. 

**Figure 1:**
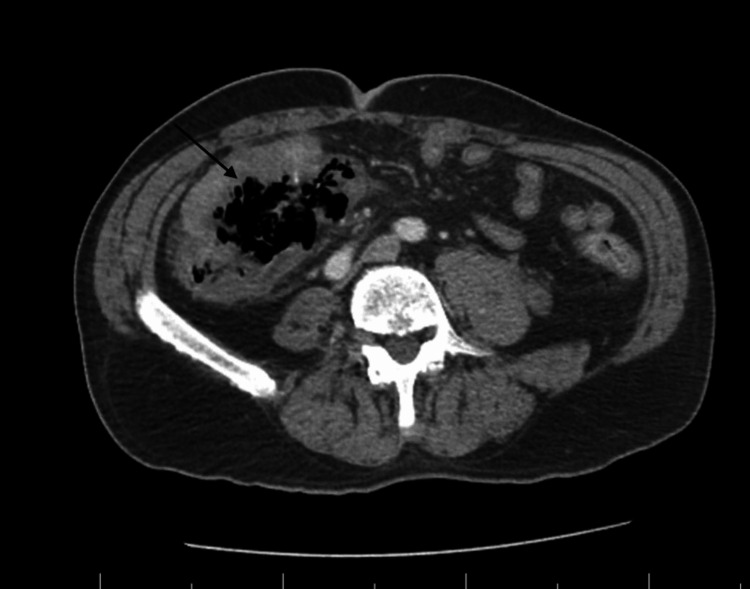
CT Abdomen/Pelvis: Arrow Points to Colon Perforation

On physical exam, the patient had significant right lower quadrant tenderness with involuntary guarding and focal peritonitis. Significant laboratory findings revealed leukocytosis 28,400/mL (absolute neutrophil count 26.13), anemia with hemoglobin of 7.4 g/dL, thrombocytosis 549,000/uL, bicarbonate 19 mEq/L, magnesium 1.7 mEq/L, and lactic acidosis 2.9 mmol/L. CT of abdomen/pelvis with intravenous (IV) contrast demonstrated marked eccentric thickening and irregularity of the proximal portion of the ascending colon with adjacent stranding and irregular cavity-like appearance containing air with multiple focal areas of free intraperitoneal air in the right mid-abdomen (Figure 1).

The patient was started on IV vancomycin and piperacillin/tazobactam and taken to the operating room for exploratory laparotomy. A large mass measuring 8.0x7.5x3.0 cm was noted within the right colon and cecum with a punctate area that was leaking air and mucous. The mass was invading the small bowel and mesentery about 5 cm proximal to the terminal ileum. A right hemicolectomy was performed taking the small bowel in an en bloc resection and taking the mesentery down to its root in order to harvest sufficient lymph nodes for staging. Primary stapled anastomosis between the colon and small bowel was made. The patient tolerated the procedure well. 

In the immediate postoperative period the patient required 4 units of packed red blood cells (PRBC). CT chest with IV contrast was performed during admission significant for two cardio phrenic lymph nodes which were likely reactive and thoracic aortic aneurysm 4.5 cm in size. The remainder of his hospital course was uneventful and the patient recovered appropriately. The patient was deemed stable for discharge on postoperative day eight. 

**Figure 2:**
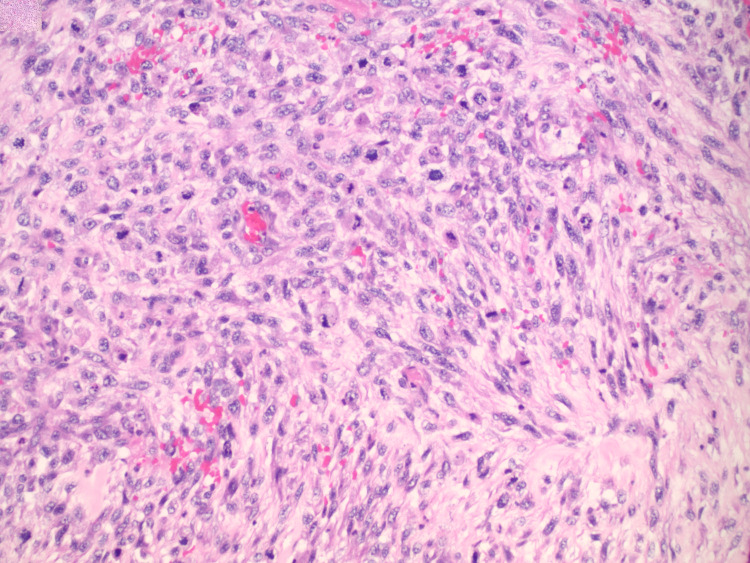
Hematoxylin and Eosin (H&E) Stain of Sarcoma

**Figure 3:**
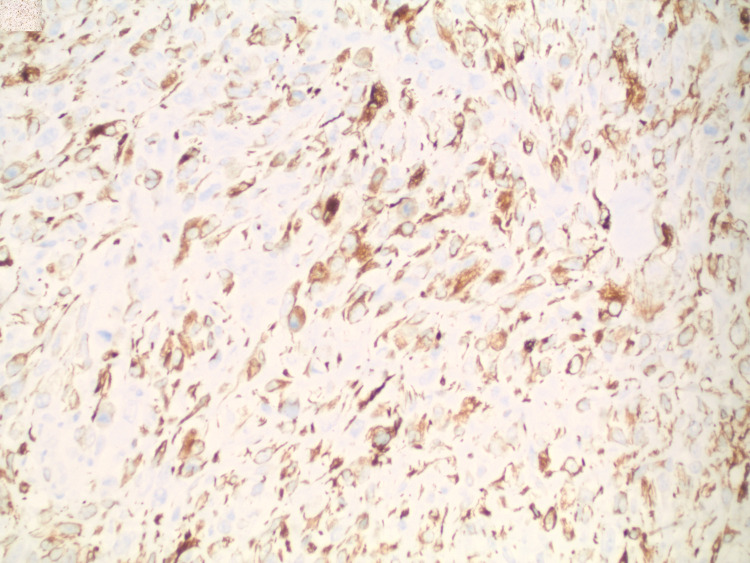
Immunostain for Desmin (brown stain)

The mass was diagnosed as high-grade leiomyosarcoma based on histologic and immunohistochemical findings. The leiomyosarcoma invaded through the cecal wall to involve the adjacent mesentery, but did not invade adjacent organs. Lymphovascular invasion was absent (15 nodes negative) and margins were negative. Microscopically the most active areas of the tumor contain 12 mitoses per 10 high power fields with necrosis involving 40% of the tumor sampled, indicating a Grade 3 tumor based on the French Federation of Cancer Centers Sarcoma Group (FNCLCC) Grading system (Figure 2). Furthermore, immunohistochemistry showed malignant spindle cells that exhibited desmin positive (Figure 3), actin negative, cluster of differentiation (CD) 117 negative, CD 34 negative, AE1/AE3 negative, s100 negative, and cytokeratin 20 (CK 20) negative. Pathologic staging T2b N0 Mx.

The specimen was re-reviewed at a tertiary care center due to uncommon pathology at a local community hospital and updated to be DDLS with myogenic and osteosarcomatous differentiation in a background of well-differentiated liposarcoma. The sarcoma also involved one of 15 lymph nodes which may have been a direct extension of the tumor onto the lymph node. Based on the new findings upon re-review, American Joint Committee on Cancer (AJCC) stage was changed to Stage III (T2b, N0, M0, GS) making this a high-grade DDLS. A multi-specialty tumor board recommended adjuvant chemotherapy with doxorubicin/ifosfamide once the patient had recovered from surgery. 

The patient presented to the emergency department a week prior to the start of chemotherapy, approximately 10 weeks from index operation, with increased abdominal pain. CT Chest/Abdomen/Pelvis was performed and was significant for development of two new metastatic nodules within the left lung apex and superior right middle lobe measuring 4 mm, extensive hepatic metastatic disease with mass in the anterior segment right hepatic lobe measuring 4.4 x 3.0 cm, and development of multiple peritoneal implant masses within the right lower quadrant and left lower quadrant with pelvic ascites. Soon thereafter, the patient was to be unable to keep the first chemotherapy appointment and was documented to be deceased a week later, approximately three months after the index operation.

## Discussion

DDLS involving and invading the colon is rare and only a few cases have been reported in the literature. This case demonstrates a mass that was based in the cecal wall and mesentery that presented as perforation and complication of metastatic disease and death within three months. 

Aggressiveness and metastatic potential are based on differentiation of the tumor. There are three main morphologic subgroups: well-differentiated/dedifferentiated, myxoid/round cell, and pleomorphic liposarcoma. DDLS is typically a high-grade sarcoma with metastatic potential that arises from well-differentiated liposarcoma, and usually presents as a large mass in the retroperitoneum. Well-differentiated liposarcoma, unlike DDLS, does not possess metastatic potential [1-4].

The majority of cases of DDLS arise from the retroperitoneum and present as gastrointestinal masses on initial diagnosis, which have been reported in the stomach, small intestine, and colon. They are often asymptomatic until the tumor grows to a significant size causing obstruction or mass effect. Evaluation is best achieved by CT or magnetic resonance imaging (MRI) [1].

Our patient presented with an extremely aggressive dedifferentiated liposarcoma of the cecal mesentery and cecal wall. A CT abdomen/pelvis was performed six months prior to index operation due to presentation with kidney stones, and at that time, there was no evidence of any abnormality of the right colon, mesentery, or retroperitoneum. Since this patient presented with a perforated colon, emergency surgery was performed. Once a cecal mass is identified, the standard of care is to perform a right hemicolectomy including the cecum, ascending colon, and hepatic flexure. The proximal and distal resection margins should be at least 5-7 cm from the tumor [5]. The colon should be removed en bloc with its associated mesentery to the origin of the primary feeding vessel. This provides regional mesenteric lymph nodes along the course of the mesenteric vessels as well as those adjacent to the colon for adequate staging [6]. Patients who present with perforated colon cancer have been found to have worse survival compared to non-perforated colon cancers after adjusting for stage and medical comorbidities. Treatment options in patients with perforation depend on the patient’s overall clinical condition and exam. Once the patient is in the operating room, hemodynamic stability, bowel viability, and peritoneal contamination determine if primary anastomosis can be performed [7]. Primary anastomosis should not be performed in the setting of hemodynamic instability, severe gross contamination, or large pressor requirements.

Surgical resection is the most common treatment option for DDLS [2-4]. Local recurrence rates vary widely, ranging between 31-83% with 20-30% distant metastatic recurrence rate within three years [1]. DDLS five- and 10-year survival rates are estimated as 57% and 40% as compared to well-differentiated liposarcoma of 100-82% respectively [8].

Postoperative treatment for liposarcoma remains controversial. Adjuvant chemotherapy limits the risk of local recurrence with a doxorubicin-based regimen, but no definite survival benefit has been established [1]. In this case, adjuvant radiation or chemotherapy was not initiated due to rapid clinical deterioration. 

## Conclusions

This case discusses a DDLS presenting with cecal perforation. There are few reported cases of DDLS of the colon in literature. In our limited review, no cases presented with a perforation. The patient underwent an emergent operation. Surgical pathology showed DDLS with local invasion. Staging CT of the chest while the patient was hospitalized showed no evidence of metastatic disease. The patient had aggressive progression of metastatic disease and rapid clinical deterioration. Adjuvant chemoradiation is recommended for treatment options, but our patient was unable to receive the first dose due to disease progression. The survival rate is low and recurrence is high. Hence, realistic treatment targets and goals of care should be established early with patients who are diagnosed with this condition.

## References

[1] Mullen JT, Delaney TF (2019). Clinical Features, Evaluation, and Treatment of Retroperitoneal Soft Tissue Sarcoma. UpToDate.com.

[2] Sawayama H, Yoshida N, Miyamoto Y (2017). Primary colonic well-differentiated / dedifferentiated liposarcoma of the ascending colon: a case report. Surg Case Rep.

[3] Hollowoa B, Lamps LW, Mizell JS (2018). Dedifferentiated liposarcoma mimicking a primary colon mass. Int J Surg Pathol.

[4] Türkoğlu MA, Elpek GÖ, Doğru V, Calış H, Uçar A, Arıcı C (2014). An unusual case of primary colonic dedifferentiated liposarcoma. Int J Surg Case Rep.

[5] Rodriguez-Bigas MA (2020). Surgical Resection of Primary Colon Cancer. UpToDate.com.

[6] Edge SB, Compton CC (2010). The American Joint Committee on Cancer: the 7th edition of the AJCC cancer staging manual and the future of TNM. Ann Surg Oncol.

[7] Daniels M, Merkel S, Agaimy A, Hohenberger W (2015). Treatment of perforated colon carcinomas-outcomes of radical surgery. Int J Colorectal Dis.

[8] Knebel C, Lenze U, Pohlig F (2017). Prognostic factors and outcomes of liposarcoma patients: a retrospective evaluation over 15 years. BMC Cancer.

